# Fatal interactions: pneumonia in bighorn lambs following experimental exposure to carriers of *Mycoplasma ovipneumoniae*

**DOI:** 10.1128/jcm.01328-24

**Published:** 2025-01-21

**Authors:** Logan K. Weyand, Brandi L. Felts, E. Frances Cassirer, Jonathan A. Jenks, Daniel P. Walsh, Thomas E. Besser

**Affiliations:** 1 Department of Veterinary Microbiology and Pathology, Washington State University 6760 , Pullman, Washington, USA; 2 Department of Natural Resource Management, South Dakota State University 2019 , Brookings, South Dakota, USA; 3 Idaho Department of Fish and Game 115870 , Lewiston, Idaho, USA; 4 US Geological Survey National Wildlife Health Center 168533 , Madison, Wisconsin, USA; Boston Children's Hospital, Boston, Massachusetts, USA

**Keywords:** bighorn sheep, *Mycoplasma ovipneumoniae*, epizootic pneumonia, chronic carriage, captive, exposure, source, pathogen transmission, genetic strain

## Abstract

We hypothesized that bighorn sheep ewes with chronic nasal *Mycoplasma ovipneumoniae* carriage are the source of infection that results in fatal lamb pneumonia. We tested this hypothesis in captive bighorn ewes at two study facilities over a 5-year period, by identifying carrier ewes and then comparing lamb fates in groups that did (exposed pens) or did not (non-exposed pens) include one or more carrier ewes. Most (23 of 30) lambs born in exposed pens, but none of 11 lambs born in non-exposed pens, contracted fatal pneumonia. In addition, surviving lambs in exposed pens showed obvious signs of respiratory disease while lambs in non-exposed pens did not. In crossover experiments, individual non-carrier ewes had lambs that experienced fatal pneumonia in years when housed in exposed pens, but not in years when housed in non-exposed pens. The results of these studies clearly associate lamb pneumonia to exposure to *M. ovipneumoniae* carrier ewes, consistent with a necessary role for this agent in epizootic pneumonia of bighorn sheep. These data specifically highlight the role of chronic *M. ovipneumoniae* carriage by some bighorn ewes in the epidemiology of this population-limiting wildlife disease.

**IMPORTANCE:**

Bighorn sheep populations, historically important in mountain and canyon ecosystems of western North America, declined precipitously following European settlement of North America and remain depressed today. One factor contributing to these declines and lack of recovery is epizootic pneumonia caused by the bacterium *Mycoplasma ovipneumoniae*. This pathogen arrived with settlers’ domestic sheep and goats and spilled over to infect bighorn sheep, a process that continues to this day. Bighorn losses from this disease include high rates of mortality (median, approaching 50%) of all ages of bighorn sheep on initial exposure, followed in subsequent years to decades by mortality largely limited to young lambs. The source of infection causing persistent lamb losses is the focus of the research described here. Conducting these studies on groups of captive bighorn sheep enabled demonstration of clear linkage between largely asymptomatic nasal carriage of *M. ovipneumoniae* by ewes and outbreaks of fatal pneumonia in lambs.

## INTRODUCTION

Bighorn sheep (*Ovis canadensis*) is an iconic North American wildlife species that experienced dramatic historical population declines, with only a limited response to restoration efforts undertaken since the first half of the 20th century (1, 2). One important barrier to restoration of bighorn sheep populations is the disease complex, epizootic pneumonia (3, 4). When this disease initially strikes previously healthy populations, all age classes are affected with a median mortality approaching 50%; subsequently, the disease is largely restricted to lambs, resulting in impaired recruitment and population recovery for years or even decades (3, 5).

The bacterial pathogen *Mycoplasma ovipneumoniae* is considered the primary causal agent associated with both all-ages and lamb-only stages of bighorn sheep pneumonia (3, 4). Early work based on aerobic cultures demonstrated bighorn sheep pneumonia to be polymicrobial and emphasized the potential significance of several well-recognized Pasteurellaceae respiratory pathogens. However, the predominant flora within affected lung tissues in most pneumonic bighorn sheep was later shown by metagenomic methods to consist of diverse obligate anaerobic bacteria rather than Pasteurellaceae. The disease-initiating role of *M. ovipneumoniae* was clarified by further metagenomic studies that showed this agent to be the predominant or sole bacterium present early in the disease course, epidemiologic studies that temporally correlated onset of pneumonia outbreaks with introduction of *M. ovipneumoniae* into previously non-infected populations, and identification of single genetic strains of *M. ovipneumoniae* within outbreaks consistent with epidemic transmission (4, 6–9).

We hypothesized that following an all-ages epizootic, *M. ovipneumoniae* can be maintained in bighorn sheep populations by persistently infected adults (chronic carriers) that show few or no signs of active respiratory disease such as nasal discharge, coughing, etc. (6, 10). Like most other *Mycoplasma* pathogens, *M. ovipneumoniae* does not persist well in the environment and is therefore transmitted primarily through direct contact (11). Alternative hypotheses, including primary roles for other pathogens, or environmental or nutritional effects, are less well supported by available data (4, 7). The chronic carriage hypothesis has been supported in free-ranging bighorn sheep when lamb pneumonia epizootics ceased followed the death or removal of all ewes colonized by *M. ovipneumoniae*, as confirmed by the lack of pathogen-specific antibodies in animals born after the death or removal of the last carrier ewes (7, 12, 13). Here, we report experiments designed to more directly test the effect of *M. ovipneumoniae* exposure from carrier ewes on lamb health and survival, as well as document the stability of *M. ovipneumoniae* carriage by ewes over time.

## MATERIALS AND METHODS

### Microbiological sampling

Swab samples (BD CultureSwabTM EZ System, Franklin Lakes, NJ, USA) were used to detect *M. ovipneumoniae* in nasal secretions. A single swab was used to sample both nares of each individual bighorn sheep, by deep insertion and rotation into one nostril followed by the same process on the other side, after which the swab was replaced in its dry sheath. The same samples were collected from all dead animals, and in addition, deep bronchial swabs were collected at necropsy to detect *M. ovipneumoniae* in the lower respiratory tract. *M. ovipneumoniae* DNA was detected by real-time polymerase chain reaction (PCR) in either the research laboratory of Thomas Besser or by the Washington Animal Disease Diagnostic Laboratory (WADDL) (14). Real-time PCR cycle threshold scores of 36 or lower were considered detections of *M. ovipneumoniae*, and scores of 40 were recorded as not detected; intermediate scores were considered indeterminate.

*M. ovipneumoniae* strains were identified after PCR detection using multi-locus sequence typing (MLST) conducted in the research laboratory of TEB or in WADDL (6, 15). Jugular venous blood samples (8–10 mL) were also collected, processed to separate serum, and 0.5- to 1-mL serum was submitted to WADDL for competitive enzyme-linked immunosorbent assay to detect *M. ovipneumoniae*-specific antibodies (16).

### Experimental animals

Study animals at Washington State University (WSU) (Table 1) included one bighorn ewe born in captivity at WSU and ewes captured in free-ranging populations at Rock Creek Montana (*n* = 5) and the Hells Canyon metapopulation near Lostine, Oregon (*n* = 7). The Rock Creek MT animals were relocated to WSU in early 2014; their population of origin had experienced a pneumonia epizootic associated with *M. ovipneumoniae* strain BHS-028 in 2009–2010 (8, 15), but all the Rock Creek MT animals tested negative for *M. ovipneumoniae* upon relocation to WSU. Later in 2014, both the Rock Creek MT- and the WSU-born ewes were involved in an experimental pneumonia epizootic after challenge with nasal washings from a naturally *M. ovipneumoniae-*infected domestic sheep (17). Three *M. ovipneumoniae* strains were detected in that epizootic: BHS-136/DS-185, BHS-137, and BHS-141. The Lostine OR ewes had experienced respiratory disease exposure at their herd of origin associated with *M. ovipneumoniae* strain BHS-024 and remained colonized by that same strain after transfer to WSU (15, 18).

**TABLE 1:** For each captive facility, the year the sheep were introduced into the study, the source herd, the prior *M. ovipneumoniae* strain exposures of the bighorn ewes, and the number of animals are indicated

Captive facility	Year	Source herd	Prior strain exposure	*N*
WSU	2018	Lostine OR (HC^*a*^)	BHS-024	7
WSU	2017	Rock Cr MT	BHS-028*^b^; BHS-136/DS185; BHS-137; BHS-141	5
WSU	2017	WSU captive born	BHS-136/DS-185; BHS-137; BHS-141	1
SDSU	2014	Asotin Cr WA (HC)	BHS-024	2
SDSU	2015	Asotin Cr WA (HC)	BHS-024	2
SDSU	2014	Black Butte WA (HC)	BHS-024; BHS-072*	8
SDSU	2014	Lostine OR (HC)	BHS-024	3
SDSU	2015	Lostine OR (HC)	BHS-024	2
SDSU	2015	Sheep Mtn ID (HC)	BHS-024	2
SDSU	2014	Snowstorm Mtns NV	BHS-055/DS-096	10
SDSU	2015	Snowstorm Mtns NV	BHS-055/DS-096	2
SDSU	2016	SDSU captive born	None	5

^*a*^ HC indicates herds within the Hells Canyon metapopulation.

^*b*^ Asterisks indicate strains known to have been historically present in the source herd, but never detected among study animals.

Study animals at South Dakota State University (SDSU) (Table 1) included bighorn ewes captured from free-ranging populations in the Hells Canyon metapopulation in Washington (*n* = 12, Asotin Creek and Black Butte populations), Oregon (*n* = 5, Lostine population), and Idaho (*n* = 2, Sheep Mountain population) and from the Snowstorm Mountains population in Nevada (*n* = 12), as well as five lambs born to the Washington-origin sheep in 2015 after relocation to SDSU. All source herds had experienced respiratory disease associated with one or more of three *M. ovipneumoniae* strains: BHS-024 and BHS-072 (WA), BHS-024 (OR and ID), and BHS-055/DS-096 (NV) (15) in their herds of origin prior to movement of these animals to SDSU; however, only BHS-024 (Hells Canyon) and BHS-055/DS-096 (Snowstorms) were detected at SDSU.

Animals were housed in facilities managed by the Animal Resources Unit of the College of Veterinary Medicine (WSU) and at the Captive Wildlife Research Facility in Brookings, South Dakota (SDSU). Captive animals were provided fresh alfalfa grass-mixed hay, pelleted soybean hulls, water, and loose mineral *ad libitum*.

Adult animals were immobilized using compounded butorphanol (0.43 mg/kg), azaperone (0.29 mg/kg) and medetomidine (0.17 mg/kg) (BAM, Wildlife Pharmaceuticals, CO, USA) administered by CO2 powered dart projectors (Pneu-dart, Williamsport, PA, USA) to obtain samples for microbiological or serological testing, or for moving among pens.

### Experimental design

Previous longitudinal studies have shown that *M. ovipneumoniae* carriage status of most individual ewes was quite stable, either consistently positive (chronic carriers) or consistently negative (non-carriers); however, the status of others (intermittent carriers) was more variable (3, 18). Our experimental design called for first classifying ewes for carrier status, commingling groups of ewes with known carrier status prior to parturition and recording lamb health and survival outcomes. Ewes were classified for carrier status based on pre-lambing *M. ovipneumoniae* PCR results: those with two detections were classified as carriers, those with no detections were classified as non-carriers, and those with one detection and one non-detection were classified as intermittent carriers (Table 2). Nasal swab samples with indeterminate range CT scores were not used for carrier classification but instead were replaced by the next earlier, non-indeterminate sample results from the same animal. In each breeding season, each pen was classified as “exposed” if one or more *M. ovipneumoniae* carriers were present, as “non-exposed” if all ewes were non-carriers, or as “uncertain” status if one or more “intermittent” *M. ovipneumoniae* shedders, but no chronic carriers were present. When possible, we executed a crossover design, where the same non-carrier ewes were placed in exposed and non-exposed pens in different years.

**TABLE 2:** Lamb fate by *M. ovipneumoniae* pen exposure status, biological year, study site, and carriage status of ewes

					Lamb fate^*c*^		
Pen status^*a*^	Year	Site	PenID	Ewe status^*b*^	P	O	S
Non-exposed	2014	SDSU	2	N3 (3)			3
Non-exposed	2016	SDSU	393	N3 (2)		1	1
Non-exposed	2017	WSU	6	N4 (4)			4
Non-exposed	2018	WSU	6	N3 (2)			2
Sub-total: Non-exposed pens					0	1	10
Exposed	2014	SDSU	7	C2 (1), N1 (1)	2		
Exposed	2015	SDSU	10/12	C3 (2), I4 (4), N1 (0), ND2 (1)	7		
Exposed	2016	SDSU	3	C1 (0), I1 (1)			1
Exposed	2016	SDSU	5	C1 (0), I1 (1), ND1 (0)	1		
Exposed	2016	SDSU	8	C2 (0), I1 (1)		1	
Exposed	2016	SDSU	12	C2 (2), I1 (0)	1	1	
Exposed	2017	SDSU	8	C2 (2)	2		
Exposed	2017	SDSU	12	C1 (1)	1		
Exposed	2017	SDSU	11	C2 (2)	2		
Exposed	2017	WSU	4	C1 (1), N1 (1)	2		
Exposed	2018	WSU	4	C1 (1), N2 (2)	2		1
Exposed	2018	WSU	2	C4 (2)	2		
Exposed	2019	WSU	4	C1 (0), N4 (4)	1		3
Sub-total: Exposed pens					23	2	5
Uncertain	2016	SDSU	400	N1 (1), I1 (1)			2
Uncertain	2017	SDSU	393	I1 (1), N1 (1)	1		1
Uncertain	2017	SDSU	400	I1 (1), N1 (0)	1		
Sub-total: Uncertain pens					2	0	3
Total all pens					25	3	18

^*a*^ Non-exposed (only non-carrier ewes); Exposed (at least one chronic carrier ewe present); Uncertain (no chronic carrier ewes but one or more intermittent ewe present).

^*b*^ Ewe status: C, chronic carrier; I, intermittent carrier; N, non-carrier; ND, not determined. Within each ewe status group, the number of ewes that produced lambs is noted parenthetically for each pen/year.

^*c*^ Lamb fate: P = pneumonia death; O = other cause of death; S = survived.

The carriage status of all WSU ewes and 27 of the SDSU ewes was established prior to the start of the lambing studies; during these studies, we sampled each ewe for detection of *M. ovipneumoniae* at least twice each year between 1 October and 15 March. Due to a 2015 pneumonia epizootic that prevented ewe chronic carrier classification (19), SDSU data from five pens in 2015 were not available for this analysis. Lambs that were stillborn or died at less than 48 hours of age were also censored. Breeding rams used for the study ewes were sourced from herds known to be exposed to the same *M. ovipneumoniae* strain(s) as the ewes whenever possible to avoid exposure to different strains (9).

### Biosecurity

To prevent human-assisted transmission of pathogens, personnel followed biosecurity protocols including (i) the installation of disinfecting foot baths at each pen gate for use immediately prior to entering and exiting each pen (SDSU only); (ii) use of pen-specific feed and water pails (both study sites); (iii) changing protective clothing between pens when handling sheep (SDSU only); and (iv) using order-of-entry where uninfected pens were entered prior to known infected pens (both study sites). Pens were assigned with consideration of the prevailing winds at each site, with uninfected pens located at the western (upwind) edge of the research facilities whenever possible. Minimum distances between carrier and non-carrier pens (15 m at SDSU, 50 m at WSU) were established to minimize the potential for airborne pathogen transmission between pens (20).

### Clinical scores

Live lambs at WSU were initially observed daily after birth and then less frequently through 75 days of age. At each observation, all respiratory disease signs exhibited by each animal during a 10 minute observation period per pen were recorded. Specific signs of respiratory disease recorded included coughing, labored respiration, watery or purulent nasal discharge, ear drooping/paresis, repetitive nose licking or head shaking, ocular discharge, lethargy, recumbency, and separation from the ewe. The number of signs observed from each animal each day was summed to determine daily clinical scores.

### Gross necropsy and histopathology

Lambs that died or were euthanized during the study were submitted to pathologists at the South Dakota Animal Disease Research and Diagnostic Laboratory in Brookings SD (2014 lambs) or to WADDL (all subsequent lambs) for complete gross necropsy and histopathology, and expert opinions regarding the cause of death.

## RESULTS

**Fig 1:**
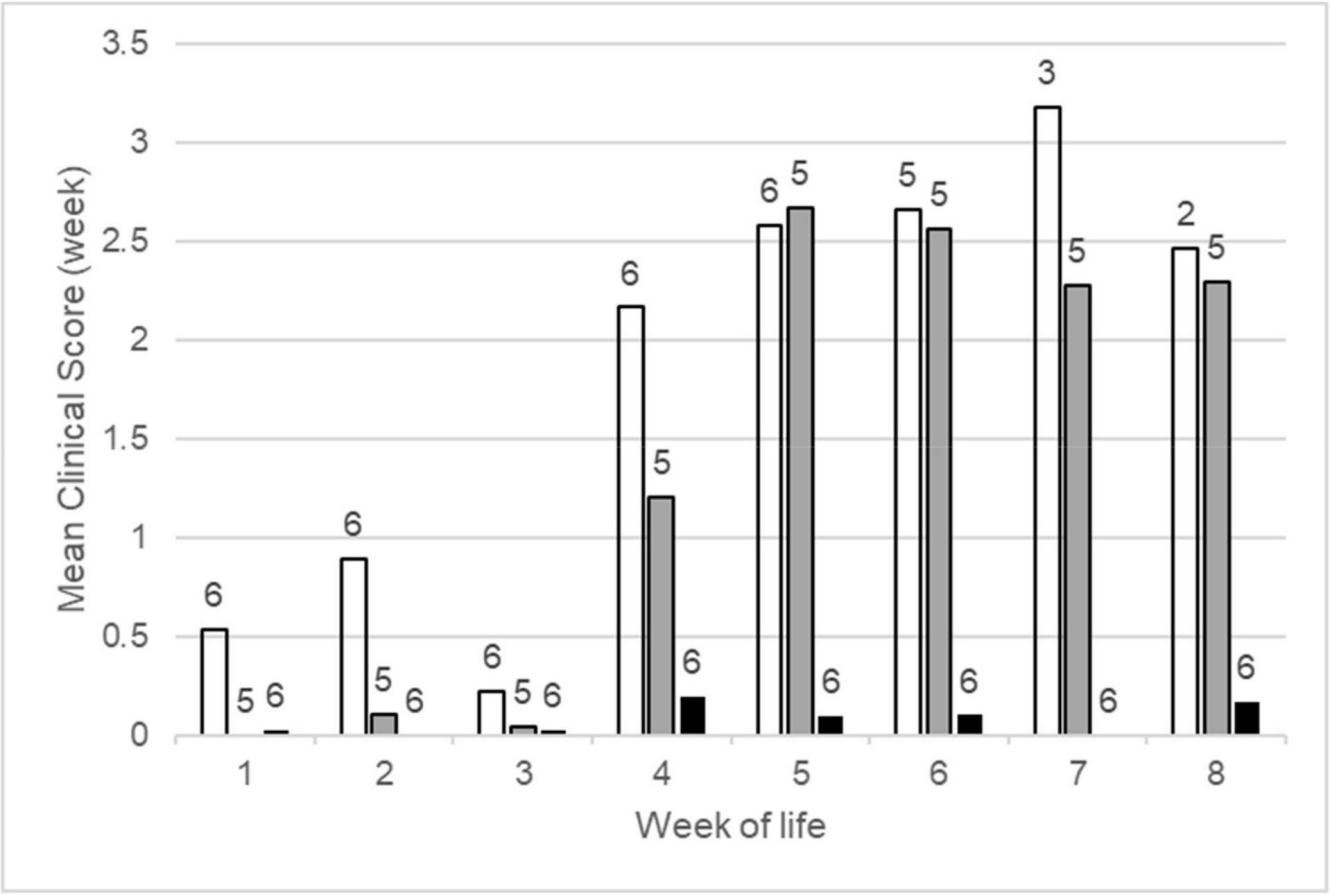
Respiratory disease signs observed in bighorn lambs at WSU during the first 8 weeks of life. Signs recorded included coughing, labored respiration, watery or purulent nasal discharge, ear drooping/paresis, repetitive nose licking or head shaking, ocular discharge, lethargy, recumbency, and separation from the ewe. Mean daily numbers of respiratory signs observed per lamb are compared for lambs born in exposed pens that died of pneumonia (open bars), lambs born in exposed pens that survived (grey-shaded bars), and lambs born in non-exposed pens (black bars). The numbers of lambs contributing to the scores are indicated above each bar. The numbers of pens in each group for each year are reported in Table 2.

The study included 46 lambs born to 33 ewes: 30 lambs born in 13 *M*. *ovipneumoniae* exposed pens, 11 lambs born in four non-exposed pens, and five lambs born in three pens with uncertain exposure (Table 2). A non-significant tendency for carrier ewes to lamb at lower rates (15 of 24 ewes, 62.5%) than non-carrier ewes (19 of 23 ewes, 82.6%) was observed. All lambs in exposed pens were observed with nasal discharge, repeated nose licking or head shaking, coughing, and separation from the dam and many also with ear paresis, labored respiration, lethargy, or recumbency. In contrast, the signs occasionally observed in lambs in non-exposed pens were limited to nasal discharge, repeated nose licking or head shaking, and ocular discharge. Of the 30 lambs born in exposed pens, 23 (77%) died with pneumonia, while pneumonia was not observed among the 11 lambs born in non-exposed pens. Five lambs were born in pens with uncertain exposure status, of which two died of pneumonia and three survived. Both the overall frequency of lambs dying of respiratory disease and the frequency of pens in which at least one lamb died of respiratory disease were clearly associated with pen exposure status (2 × 3 Fisher’s exact tests, *P* = 0.000006 and *P* = 0.004, respectively) (21). Numerous clinical signs of respiratory disease were also evident in surviving lambs in exposed pens, while lambs in non-exposed pens exhibited few (*N* = 2 lambs) or no (*N* = 4 lambs) signs of respiratory disease (Fig. 1). *M. ovipneumoniae* infection in the five surviving lambs born in exposed pens was confirmed by PCR. All became PCR-negative by 1 (three lambs) or 2 (two lambs) years of age. Surviving lambs in non-exposed pens were negative for detection of *M. ovipneumoniae* by PCR.

The occurrence of fatal lamb pneumonia was also associated with the lambing status of the chronic carrier ewes themselves in exposed pens: in four exposed pens in which the carrier ewe(s) did not give birth, two lambs died with pneumonia, one lamb died of other causes (contagious ecthyma and malnutrition/starvation) at 78 days of age, and four lambs survived (4/7, 57%). In comparison, in the nine exposed pens in which one or more carrier ewes did give birth, 21 lambs died with pneumonia, one died of other causes at 9 days of age, and one lamb survived (1/22, 5%); lamb survival in exposed pens was associated with carrier ewe lambing status (2 × 3 Fisher’s exact test, *P* = 0.003).

In crossover studies at WSU, five non-carrier ewes gave birth in exposed and non-exposed pens in different years (Table 2). Cumulatively, these ewes had six lambs in years when placed in non-exposed pens, all of which survived (6/6, 100%), and seven lambs in years when placed in exposed pens, of which three died of pneumonia and four survived (4/7, 57%). Three of these four surviving lambs were born in pens in which the carrier ewe did not lamb.

Serum antibodies specific for *M. ovipneumoniae* were evaluated in study lambs, either at necropsy or at >8 weeks of age. These antibodies were detected in most (16/18) tested lambs born in exposed pens but were not detected in any of the six tested lambs born in non-exposed pens. The two exposed pen lambs in which antibody was not detected both died of pneumonia, at 39 and 116 days of age, in pens in which the carrier ewe(s) lambed. As expected from their documented exposures to *M. ovipneumoniae* before and during these studies, *M. ovipneumoniae*-specific antibodies were detected in most (45/47) of the ewes tested.

Overall, severe bronchopneumonia or pleuropneumonia was the predominant attributed cause of lamb death or illness requiring euthanasia (*N* = 22/25, 88%) in exposed pens. One lamb’s tissues were too severely autolyzed to enable the pathologist to report a cause of death, although suppurative bronchiolitis and severe atelectasis were observed. Other causes of lamb deaths included two lambs in exposed pens that died at 9 and 78 days of age of starvation/malnutrition following deaths of their dams. Finally, one lamb in a non-exposed pen died at 96 days of age of starvation/malnutrition associated with severe contagious ecthyma.

**Fig 2:**
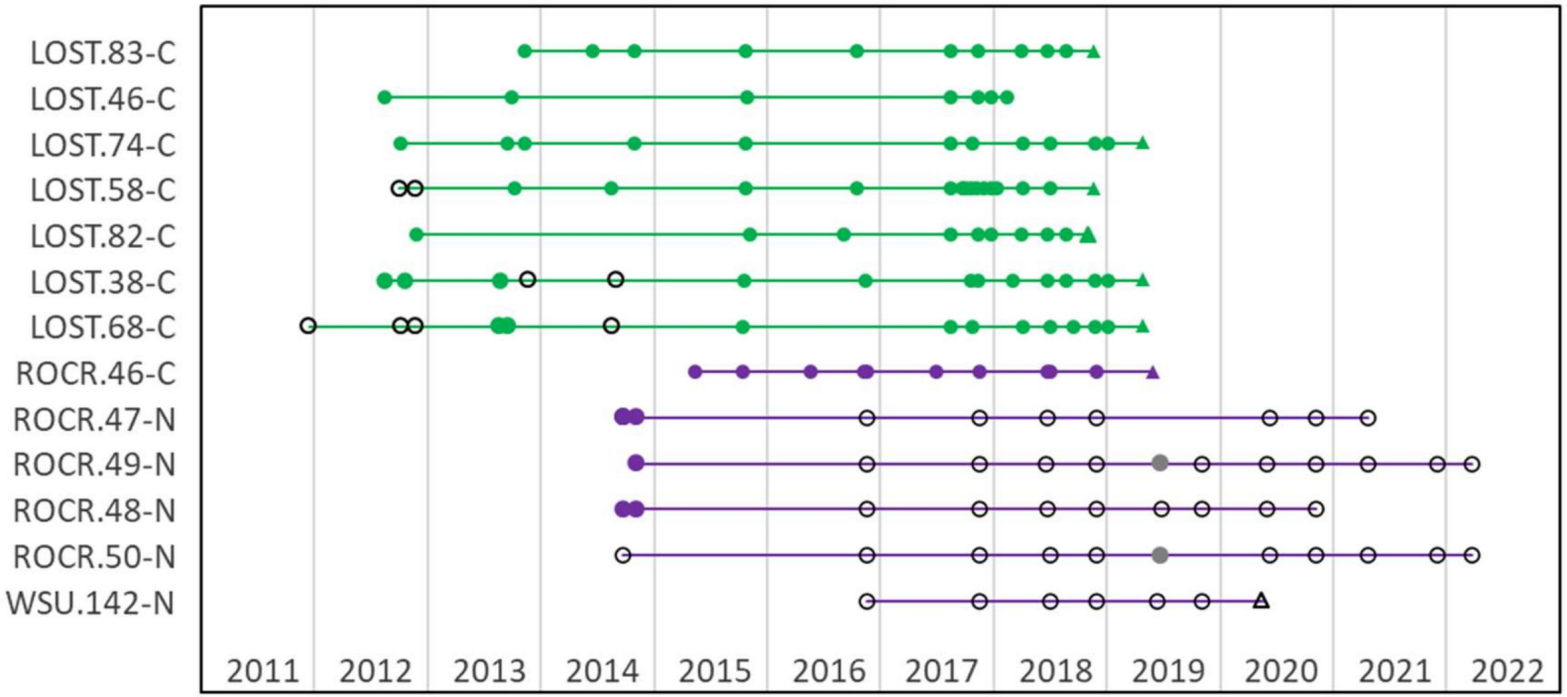
*M. ovipneumoniae* longitudinal testing of 13 ewes at WSU, 2011–2022. *Y*-axis labels indicate animal origins (LOST, Lostine OR; ROCR, Rock Creek MT; WSU, WSU captive herd), ewe eartag numbers, and ewe carrier status at the start of the captive studies (C, carrier; N*,* Non-carrier). Symbols represent results of PCR testing for *M. ovipneumoniae*: filled = detected; unfilled = not detected. Grey-filled symbols indicate indeterminate PCR results. Colors represent pre-study strain exposures (lines) and strain types identified within samples (symbols): green, BHS-024; purple, BHS-136/DS-185, BHS-137, or BHS-141. Round symbols are samples from live sheep and triangular symbols are necropsy samples.

**Fig 3:**
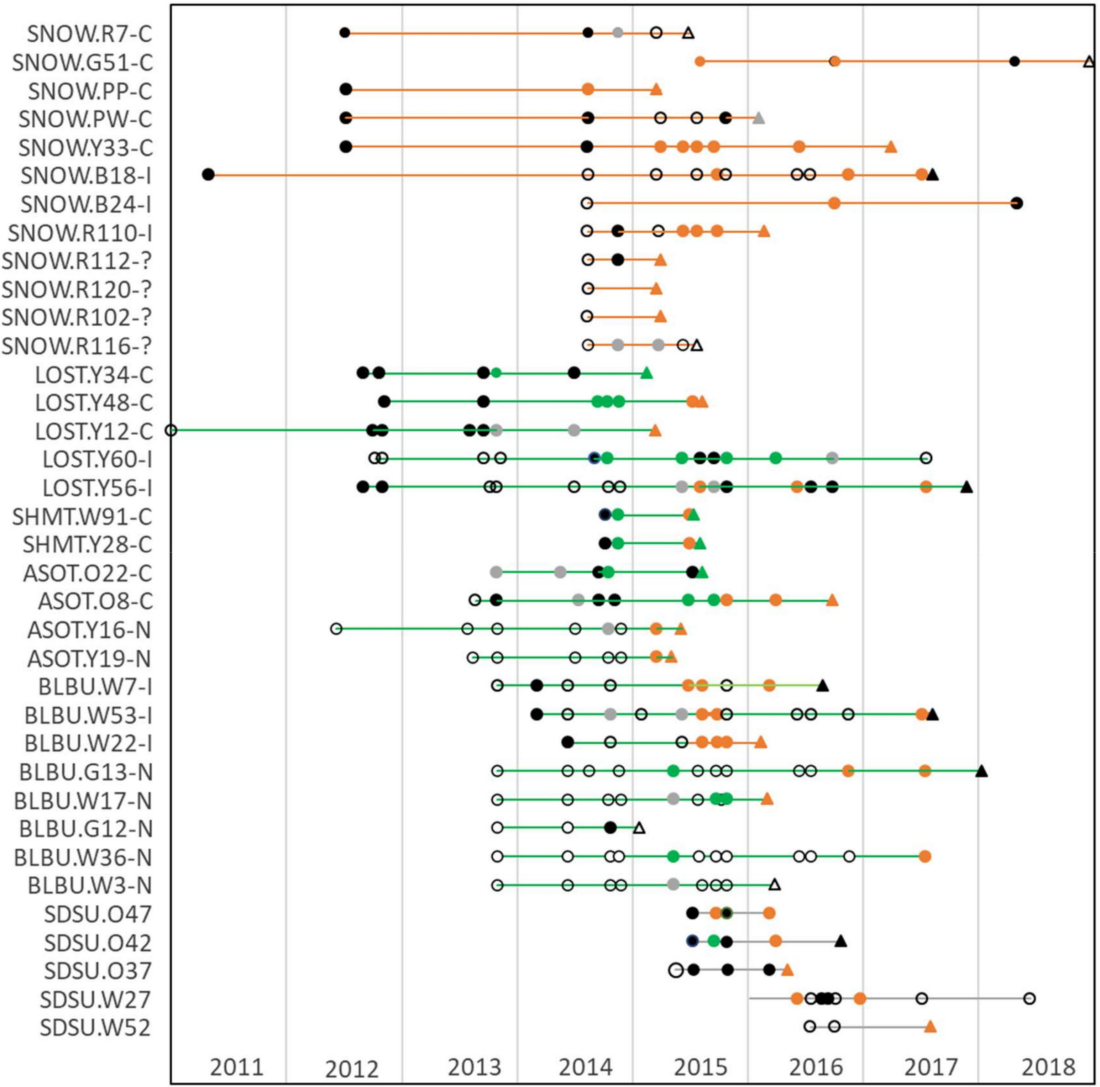
*M. ovipneumoniae* longitudinal testing of 36 ewes at SDSU, 2011–2018. *Y*-axis labels indicate animal source (SNOW, Snowstorm Mtns NV; LOST, Lostine OR; SHMT, Sheep Mtn ID; ASOT, Asotin Cr WA; BLBU, Black Butte WA; SDSU, captive born, ewe eartag numbers, and ewe carrier status at the start of the captive studies (C, carrier; N, Non-carrier; ?, unknown). Symbols represent results of real-time PCR testing for *M. ovipneumoniae*: filled, detected; unfilled, not detected. Grey-filled symbols indicate indeterminate PCR results. Colors indicate pre-study strain exposures (lines) and strain types detected within samples (symbols): orange, BHS-055/DS-096; green, BHS-024; black, strain type not determined). Round symbols are samples from live sheep, and triangular symbols are necropsy samples.

The ewes in this study had diverse patterns of *M. ovipneumoniae* carriage over time. The 41 ewes that were longitudinally sampled prior to the start of the study included 20 chronic carriers, eight intermittent carriers, and 13 non-carriers. At WSU, these patterns remained stable (Fig. 2), but at SDSU some ewes’ carriage status changed after the 2015 epizootic (Fig. 3).

The WSU Lostine ewes were all chronic carriers of strain BHS-024 throughout the commingling studies, and ewe MT46 was consistently the only chronic carrier in the WSU/Rock Creek MT ewe group (Fig. 2). At SDSU, two strains were detected: BHS-055/DS-096 (Snowstorm Mountains NV) and BHS-024 (Hells Canyon populations Lostine OR, Sheep Mountain ID, and Asotin Creek and Black Butte WA). The 2015 acute disease epizootic at SDSU was primarily associated with pen-to-pen transmission of BHS-055/DS-096 (Fig. 3) (19), resulting in its detection in many ewes (14/19, 74%) that did not originate from the Snowstorm Mtns NV source herd. These cross-strain infections sometimes coincided with changes in the previously determined carriage or non-carriage patterns of the ewes: for example, chronic detection of BHS-055/DS-096 was seen in some ewes that had previously shed BHS-024 (LOST.Y48, ASOT.O8) or that had previously been non-carriers (LOST.Y48, ASOT.O8,ASOT.Y16, ASOT.Y19, BLBU.W7, BLBU.W53, BLBU.W22. and BLBU.G13) (Fig. 3).

## DISCUSSION

We observed a clear association between ewe *M. ovipneumoniae* carriage status and the occurrence of fatal lamb pneumonia within pen cohorts of captive bighorn sheep. Fatal lamb pneumonia occurred at high frequency in pens containing known carrier ewes, sometimes occurred in pens with uncertain exposure, and never occurred in pens lacking carriers. In addition, surviving lambs born in exposed pens showed severe signs of respiratory disease comparable to those shown by lambs that subsequently died of pneumonia, consistent with the occurrence of (non-fatal) pneumonia. These results are consistent with the hypothesis that carrier ewes are an important source of infection resulting in lamb pneumonia epizootics and also consistent with the emerging results from free-ranging bighorn sheep populations where removal or loss of all identified chronic carrier ewes is associated with improved lamb survival and recruitment (7, 12, 13).

An important role for *M. ovipneumoniae* transmission among lambs within nursery groups has been previously suggested (3, 4). Bighorn ewes raise their offspring in nursery groups where there is opportunity for frequent lamb-to-lamb contact. Pathogen transmission among juveniles may be a significant route of infection in this highly social group-living species (3, 4, 22). Lambs born to chronic carrier ewes may represent an especially important link in the chain of transmission of *M. ovipneumoniae*: we observed a higher rate of fatal pneumonia in pens where carrier ewes birthed live lambs. We speculate that this effect may be the result of early transmission of *M. ovipneumoniae* from carrier ewes to their own lambs, leading to more rapid transmission to the other lambs in each nursery group and thus lamb infections at (younger) ages more susceptible to fatal disease. In comparison, direct transmission from a carrier ewe to other ewes’ lambs may be relatively inefficient, delaying introduction of the pathogen into the lamb nursery group to a time when they had increased age-related resistance to fatal disease (22).

Given the important role of ewes chronically carrying *M. ovipneumoniae* in the occurrence of epizootic lamb pneumonia, it is important to understand the host, pathogen, and environmental factors that may result in chronic carriage. Most of the bighorn ewes in these studies had carriage patterns established prior to translocation to captivity, and these patterns were often maintained after translocation. For example, Lostine OR was the source of 11 chronic and two intermittent carrier ewes across the two study sites; these classifications remained stable throughout the studies at the WSU site and prior to the 2015 epizootic at SDSU. Black Butte and Asotin Cr WA populations were the source of five and two non-carrier ewes, respectively, that initially maintained that status after translocation to captivity at SDSU.

We have previously reported that bighorn sheep exposed to (and apparently immune to) one *M. ovipneumoniae* strain are nevertheless susceptible to disease when infected with a different strain (9), although it is currently unknown whether this susceptibility applies to all other strains. The 2015 SDSU BHS-055/DS-096 strain epizootic, described in separate publication (19), is another example of this susceptibility to disease when exposed to a new strain. Here, we can add that exposure and infection with novel strains can also apparently affect the ewes’ carrier status. Following the 2015 epizootic, BHS-055/DS-096 became the most frequently detected strain in all SDSU bighorn ewe groups, including those not previously exposed to this strain.

*M. ovipneumoniae* carriage patterns of some ewes changed during and after the 2015 SDSU epizootic, compared to their pre-epizootic status. For example, LOST.Y56 (pre-epizootic an intermittent shedder of strain BHS-024) and ASOT.O8 (pre-epizootic a chronic carrier of BHS-024) both became chronic carriers of BHS-055/DS-096 after the epizootic. In contrast, carriage patterns in WSU ewes remained stable in both Lostine ewes (exposed to a single strain type) and WSU/Rock Creek ewes (simultaneously exposed to three strain types prior to the beginning of these commingling studies). The reasons for the differences in carriage pattern stability at the two study sites are unknown, but one possible explanation is the timing of exposures: the synchronous multi-strain infection exposure at WSU occurred more than 2 years prior to the start of these commingling studies, while the SDSU commingling studies were entirely completed less than 2 years after their cross-strain exposure. In addition, other unknown host or agent genetic factors could plausibly affect the development of strain-specific immune responses and stable carriage patterns. Unstable carriage patterns would be expected to adversely affect “test-and-remove” strategies for reducing lamb pneumonia losses in free-ranging populations that rely on identification and removal of chronic carrier animals, so these strategies should be applied cautiously, perhaps especially in populations that have had recent exposures to multiple *M. ovipneumoniae* strains.

*M. ovipneumoniae* strain typing based on sequencing PCR-amplified target loci is presumed to identify only the numerically predominant strain type when mixed infections occur within single animals (19). Therefore, detection of different strain types in individual ewes from samples taken at short intervals (e.g., in this study, ewes SHMT.W91 and SHMT.Y28 at SDSU in 2015) is consistent with simultaneous carriage of these two strains. At WSU, strains BHS-136/DS-185 and BHS-141 were both identified in ewe MT46 in different samples during the study, and strain BHS-137 was detected in a study lamb in 2018. These strains had all been identified during the 2014 experimental pneumonia outbreak that preceded this study, and so these results are consistent with simultaneous carriage of all three strains by MT46 over a period of several years.

This study had several limitations. First, obtaining research animals of known *M. ovipneumoniae* strain exposure and carriage status was expensive and difficult. The resulting relatively small sample sizes could have resulted in lack of sufficient power to test our hypothesis, but fortunately the effect of ewe *M. ovipneumoniae* carriage was sufficiently strong across source populations and strain types to provide a convincingly robust association. However, we acknowledge that it may not be true for other herds and strains not represented in our study. Second, the inability of the current MLST strain typing method to characterize minority strain(s) limited our ability to identify animals simultaneously carrying multiple strains.

In conclusion, these captive studies provide support for the hypothesized role of chronically *M. ovipneumoniae*-infected bighorn ewes in the occurrence of lamb losses during lamb pneumonia epizootics in free-ranging populations. The increased lamb mortality that occurred when chronic carrier ewes themselves produced lambs suggests that ewe-to-lamb and subsequent lamb-to-lamb transmission are important epidemiological processes, but this was not directly tested in this study. Rapid initial pathogen transmission from carrier ewes to their lambs may promote earlier introduction of the pathogen into the rest of the lamb nursery group, as also suggested by observations in free-ranging populations (3, 22). In captivity at least, carriage patterns of *M. ovipneumoniae* by ewes may require 2 or more years to stabilize following infection or exposure.

## Data Availability

DNA sequences of MLST alleles for representative examples of the strains BHS-024 and BHS-055/DS-096 are located in GenBank accessions MN019238, MN028055, MN037072, MN036653 (BHS-024); MN019186, MN028003, MN037020, MN036601 (BHS-055/DS-096), Allele DNA sequences for BHS-136/DS-185, BHS-137, and BHS-141 are located in GenBank accessions PQ642034-PQ642046 (IGS), PQ638373-PQ638385 (16S), and PQ609248-PQ609270 (*rpoB* and *gyrB*). Data used to construct tables and figures may be accessed at https://doi.org/10.5066/P14MTUBA.
