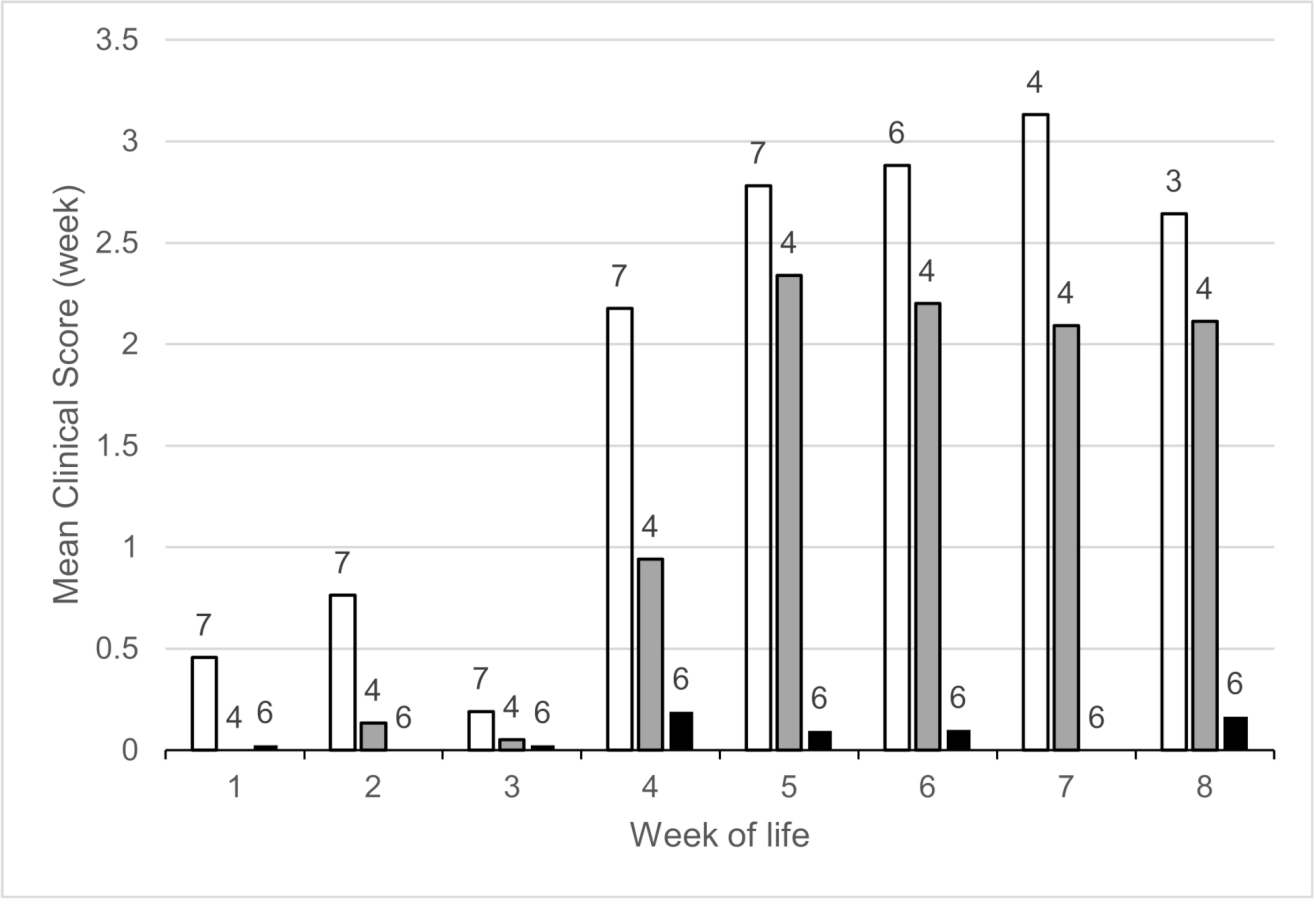# Correction for Weyand et al., “Fatal interactions: pneumonia in bighorn lambs following experimental exposure to carriers of *Mycoplasma ovipneumoniae*”

**DOI:** 10.1128/jcm.00426-25

**Published:** 2025-06-10

**Authors:** Logan K. Weyand, Brandi L. Felts, E. Frances Cassirer, Jonathan A. Jenks, Daniel P. Walsh, Thomas E. Besser

## AUTHOR CORRECTION

Volume 63, no. 2, e01328-24, 2025, https://journals.asm.org/doi/10.1128/jcm.01328-24. Page 6, Fig. 1: While preparing Fig. 1, we erroneously included one lamb in the ‘exposed survived’ group that should have been in ‘exposed died of pneumonia’. The correct Fig. 1 should appear as shown in this correction.

**Fig 1:**